# Nocturnal Dim Blue Light Is Associated with Splenic Immune Dysregulation and Altered CORT-GR Signalling in High-Fat-Diet-Fed Mice

**DOI:** 10.3390/antiox15070800

**Published:** 2026-06-26

**Authors:** Huairuo Shi, Qingyun Guan, Zixu Wang, Jing Cao, Yulan Dong, Yaoxing Chen

**Affiliations:** State Key Laboratory of Veterinary Public Health and Safety, College of Veterinary Medicine, China Agricultural University, Haidian, Beijing 100193, China

**Keywords:** dim blue light, splenic immune dysfunction, corticosterone, glucocorticoid receptor, redox-inflammatory homeostasis

## Abstract

Artificial light at night (ALAN) has emerged as a pervasive environmental stressor that disrupts immune homeostasis. This study examined the association between nocturnal dim blue light (dBL) exposure and splenic immune alterations, with particular attention to the corticosterone-glucocorticoid receptor, or CORT-GR, signalling pathway in a high-fat diet-fed mouse model. Male C57BL/6 mice were exposed to dBL (~5 lx) during the dark phase for 12 weeks while maintained on a high-fat diet (HFD). Chronic dBL exposure was associated with splenic atrophy, impaired splenocyte proliferative responses, elevated circulating CORT, and increased splenic GR expression. dBL exposure also coincided with increased NF-κB activation, reduced Nrf2/HO-1 signalling, oxidative stress, and cytokine imbalance in the spleen. Furthermore, in an independent pharmacological cohort, inhibition of CORT synthesis or GR signalling partially attenuated these alterations. Together, these findings suggest that, within an HFD-fed mouse model, dBL exposure is associated with splenic redox-inflammatory imbalance and impaired proliferative responses, a process to which dysregulated CORT-GR signalling appears to contribute.

## 1. Introduction

As the pivotal environmental cue, light not only provides visual perception for organisms but also serves as an important regulator force regulating physiological homeostasis and behavioural circadian rhythms [1]. In mammals, light exposure exerts precise control over critical physiological processes, including the sleep–wake cycle, body temperature fluctuations, metabolic rates, and hormone secretion via neuroendocrine pathways [2,3,4]. Crucially, light’s regulatory effects on organisms are wavelength-dependent, with distinct visible wavelengths eliciting markedly different biological responses based on specific photoreceptor sensitivities [5,6]. Among these, short-wavelength blue light within the 400–480 nm range possesses higher single-photon energy and exerts the most potent stimulatory effect on intrinsically photosensitive retinal ganglion cells (ipRGCs), making it the most sensitive spectral component for regulating the biological clock [7,8]. With the proliferation of modern lighting technologies, exposure to unnatural blue light during nocturnal hours has become increasingly prevalent, highlighting the need for a clearer understanding of its systemic biological consequences.

The phenomenon of artificial light at night (ALAN) has emerged as a significant contributor to sleep disorders, metabolic dysregulation, and cardiovascular diseases in both human populations and experimental models [9,10,11]. Within the broad spectrum of ALAN, dim artificial light at night (dLAN), typically characterised by low-level illuminance (~5 lx), represents a pervasive environmental stressor in modern urban settings. Consequently, its impact has progressed from localised circadian impairment to generalised systemic immune imbalance observed in mammalian models [12,13]. As the important organ of peripheral immune defence, the spleen orchestrates essential functions for immune cell development, localisation, and the initiation of systemic responses [14,15]. Maintenance of splenic redox balance and inflammatory microenvironmental homeostasis provides an important basis for effective immune surveillance [16,17]. Therefore, understanding how environmental light stress translates into splenic functional impairment is of profound clinical relevance.

Despite this broad consensus regarding systemic immune impairment, the specific role of ALAN as an environmental stressor affecting the peripheral immune system remains largely unexplored. Indeed, although the impact of nocturnal light exposure on the central nervous system has garnered considerable attention, the splenic response to nocturnal blue light remains poorly understood. Research indicates that exposure to specific wavelengths of blue light may compromise immune defences by modulating the neuroendocrine-immune axis, yet the precise molecular regulatory pathways involved remain unclear [18,19].

Environmental light stress may be associated with peripheral organ dysfunction through neuroendocrine-immune signalling. Exposure to unnatural light spectra is known to disrupt circadian synchrony within the hypothalamic–pituitary–adrenal (HPA) axis, contributing to aberrant nocturnal elevations of the core endocrine molecule corticosterone (CORT) [20,21,22]. As a key signal regulating immune responses, the rhythmic secretion of CORT underpins the maintenance of systemic homeostasis [23,24]. Accordingly, the present study hypothesises that nocturnal dim blue light (dBL) exposure is associated with impaired splenic immune homeostasis under an HFD background. Specifically, we hypothesise that the spleen responds to aberrant CORT signals via its highly expressed glucocorticoid receptor (GR), accompanied by changes in GR expression, NF-κB-related inflammatory signalling, and Nrf2/HO-1-associated antioxidant responses [25,26].

Despite growing evidence that light at night perturbs circadian, endocrine, and immune homeostasis, previous studies have predominantly focused on the adverse physiological impacts of high-intensity light exposure [27,28,29]. Consequently, it remains unclear whether low-illuminance, wavelength-specific nocturnal light exposure is associated with alterations in the splenic immune microenvironment and which downstream pathways are most strongly implicated. This gap is particularly relevant because dim environmental blue light is a common component of urban nocturnal lighting, yet its biological effects may not be adequately captured by illuminance alone.

The strategic administration of wavelength-specific lighting is increasingly utilised to investigate and modulate physiological and circadian responses across various species, such as recent applications of customised light-emitting diode (LED) systems in equine models [30]. Building upon this broader cross-species relevance and to address the critical lack of understanding regarding low-illuminance photic stress, our study explores the wavelength-dependent splenic responses to dLAN under under an HFD background. Here, using a chronic nocturnal light-exposure model in male C57BL/6 mice maintained on a standardised high-fat diet (HFD) background [31,32,33], we tested whether 5 lx dim blue light was associated with more pronounced splenic alterations than dim white, green, or red light and whether these changes were associated with altered corticosterone signalling. We further examined whether pharmacological inhibition of corticosterone synthesis or GR signalling attenuates the observed phenotype and whether NF-κB and Nrf2/HO-1 pathways change in parallel with these alterations.

## 2. Materials and Methods

### 2.1. Animals and Treatment

**Figure 1:**
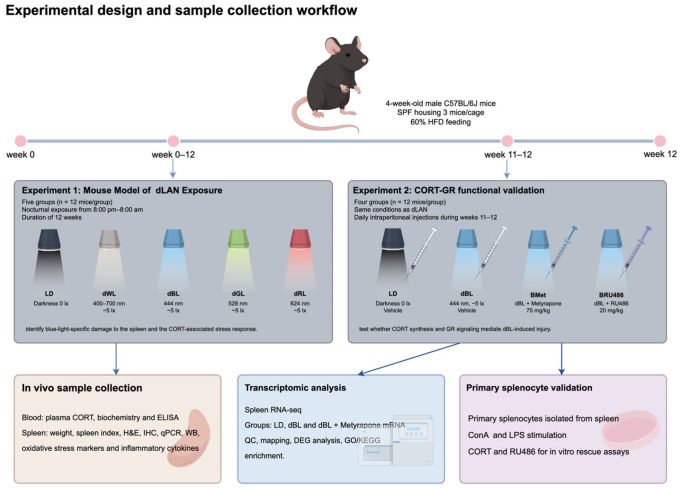
**Experimental design and workflow**. All mice were maintained on a high-fat diet for 12 weeks. Experiment 1 evaluated the effects of nocturnal monochromatic light exposure (8:00 p.m. to 8:00 a.m.) to identify wavelength-specific splenic alterations and stress responses. Experiment 2, an independent pharmacological cohort, examined the involvement of CORT-GR signalling using pharmacological inhibitors administered via daily intraperitoneal injections during weeks 11 to 12. Downstream assessments included in vivo analyses, transcriptomic sequencing, and in vitro assays in primary splenocytes. LD: control group exposed to the standard light–dark cycle; dWL: dim white light group; dBL: dim blue light group; dGL: dim green light group; dRL: dim red light group; BMet: dim blue light with Metyrapone group; BRU486: dim blue light with RU486 group.Created with Figdraw (https://www.figdraw.com/).

All three-week-old male C57BL/6J mice (Charles River Co. Ltd., Beijing, China) underwent a one-week acclimatisation period prior to the commencement of the experimental protocols. Throughout both the adaptation and subsequent experimental periods, all mice were kept in a specific pathogen-free (SPF) environment and housed three per cage. During adaptation, mice were fed a standard diet (4% of energy from fat; Charles River Co. Ltd.) and maintained on a 12 h light/12 h dark cycle (lights on at 08:00, off at 20:00). Daytime illumination was provided by white LEDs (6500 K correlated colour temperature, 400–700 nm, ~150 lx), followed by complete darkness (0 lx) during the night. The overall experimental design and workflow are shown in Figure 1.

### 2.2. Mouse Model of Dim Artificial Light at Night (dLAN) Exposure

Following the one-week adaptation, the animals were assigned to five experimental cohorts (*n* = 12 per group) using a computerised random number generator to minimise allocation bias. To implement randomised allocation and blinded endpoint assessment, an independent researcher who was not involved in daily animal handling, tissue collection, or downstream molecular analyses performed the cohort allocation and encoded the experimental light chambers. Consequently, the primary investigators responsible for conducting physiological assessments, transcriptomic sequencing, and cellular assays remained blinded to group allocation until the primary statistical analyses had been completed.

Concurrently, all animals were transitioned to a long-term high-fat diet (HFD; 60% of energy from lipids; Beijing HFK Bioscience Co., Ltd., Beijing, China) for the remainder of the study. The detailed nutritional composition and ingredient formulation of the HFD, including macronutrient distribution, fat source, energy density, and manufacturer information, are provided in Supplementary Table S1. This dietary regimen was selected to provide a consistent metabolic background relevant to contemporary dietary imbalances. The HFD was used as a fixed dietary background rather than as an experimental factor, allowing wavelength-dependent responses to nocturnal light exposure to be compared under a consistent metabolic condition.

All mice were exposed to standard white LED light (~150 lx) during the daytime photoperiod (08:00 to 20:00). For the nocturnal scotoperiod (20:00 to 08:00), the cohorts were assigned to the following specific lighting conditions: (1) LD control group: exposed to a standard light–dark cycle (12 h illumination at ~150 lx/12 h darkness at 0 lx); (2) dWL group: dim white light (6500 K, 400–700 nm, ~5 lx); (3) dBL group: dim blue light (peak wavelength 444 nm, ~5 lx); (4) dGL group: dim green light (peak wavelength 528 nm, ~5 lx); (5) dRL group: dim red light (peak wavelength 624 nm, ~5 lx). The spectral characteristics of each nocturnal light condition were defined by the colour temperature or peak wavelength, wavelength range where applicable, and measured cage-level illuminance. To ensure uniform cage-level illumination, LED strips were symmetrically mounted, and illuminance was measured daily at multiple cage corners using a calibrated photometer. In addition, all cage positions were rotated each day to minimise spatial photic bias.

This 12-week regimen of nocturnal light exposure and concurrent HFD feeding was strictly maintained across all cohorts. This extended exposure timeframe was selected to establish a chronic environmental stress model, integrating our laboratory’s previous protocols with independent foundational studies [34,35,36]. Moreover, this period precisely aligns with the standard timeframe required for establishing stable metabolic alterations under a uniform HFD baseline [37,38]. Comprehensive feeding and body-weight trajectories under this specific dual-stress paradigm have been extensively characterised in our previous studies [39]. Importantly, as the present study was not designed to test the independent effect of diet itself, the absence of a standard diet control limits causal attribution to nocturnal light exposure alone. While this synchronised design allows for the comparison of wavelength-dependent light responses under a consistent metabolic burden, it cannot disentangle the independent effects of the HFD, the specific light spectrum, or their potential interaction. At the end of the experimental period, five animals from each group (*n* = 5) were randomly selected by the aforementioned independent investigator for in vivo tissue collection, while the remaining animals were allocated for independent parallel cell extractions and in vitro assays.

### 2.3. Pharmacological Intervention with CORT Inhibitors and GR Antagonists

Following the adaptation period, an independent cohort of four-week-old male C57BL/6J mice was randomly divided into four groups (*n* = 12): (1) LD; (2) dBL; (3) dBL + Metyrapone (BMet); (4) dBL + RU486 (BRU486). The feeding conditions and light exposure parameters were identical to those described above.

Starting from the 11th week of exposure and continuing through the end of the 12th week (a 2-week intervention period), mice in the treatment groups received daily intraperitoneal injections of either the CORT synthesis inhibitor Metyrapone (75 mg/kg body weight; HY-B1232, MCE, Monmouth Junction, NJ, USA) or the GR antagonist RU486 (20 mg/kg body weight; HY-13683, MCE). Both pharmacological agents were dissolved in a vehicle solution comprising 3% DMSO and 97% saline containing 20% sulfonyl-β-cyclodextrin. To strictly mitigate the confounding effects of handling stress, the LD and dBL control groups received parallel daily intraperitoneal injections of an equivalent volume of the vehicle solvent. Furthermore, all procedures were executed rapidly by the same experienced investigator at a fixed time daily, ensuring that injection-induced stress was uniformly standardised across all experimental cohorts. Following the 12-week modelling period, five mice per group (*n* = 5) were randomly selected for tissue harvest.

### 2.4. Sample Collection and Tissue Preparation

To strictly control for the circadian fluctuations of endogenous hormones, all blood and tissue sampling procedures were consistently commenced at 08:00 (ZT0). Mice were anaesthetised with an intraperitoneal injection of sodium pentobarbital (50 mg/kg body weight), followed by blood collection via the retro-orbital sinus. The collected blood was immediately mixed with 1% sodium heparin for anticoagulation and centrifuged for 10 min at 2000× *g* (4 °C) to isolate plasma, which was subsequently stored for blood biochemistry and ELISA assays. Following blood collection, the anaesthetised mice were immediately euthanised via cervical dislocation, and the spleen tissues were promptly harvested and weighed. To assess the macro-morphological changes, the spleen index was calculated using the following equation:

*Spleen Index = Spleen Weight (* mg *)/Body Weight (* g *)*

The collected spleens were then allocated for distinct downstream applications. A fraction was freshly processed for primary splenocyte isolation, while the remaining tissue was bisected, with one half fixed in 4% paraformaldehyde for morphological evaluation and the other half snap-frozen in liquid nitrogen and stored at −80 °C for subsequent molecular analyses.

### 2.5. Haematoxylin-Eosin (H&E) Staining

Freshly collected spleen tissues were dehydrated through a graded ethanol series, cleared in xylene, and subsequently embedded in paraffin blocks. Sections 5 μm thick were prepared and mounted on slides. The sections were then dewaxed with xylene, rehydrated through a graded ethanol series, and stained with H&E. Following staining, sections were dehydrated using a graded ethanol series, cleared with xylene, and finally mounted with neutral resin. Sections were observed and photographed using an Olympus microscope (BX51, Olympus, Tokyo, Japan). Five sections were selected per group, and representative images were captured from five randomly selected fields for each section. Within each field image, ImageJ software (version 1.54p; National Institutes of Health, Bethesda, MD, USA) was employed to quantify the relative areas of splenic white and red pulp.

### 2.6. Immunohistochemistry (IHC) Staining

Paraffin-embedded spleen sections (5 µm thick) were deparaffinised in xylene, rehydrated through a graded ethanol series, and subjected to antigen retrieval. Non-specific binding was blocked with goat serum (C0265, Beyotime, Shanghai, China) for 1 h, followed by overnight incubation with the primary antibodies (PCNA polyclonal antibody, 10205-2-AP, 1:300, Proteintech, Rosemont, IL, USA; NF-κB p-p65 polyclonal antibody, 82335-1-RR, 1:500, Proteintech) at 4 °C. After a warming period, the sections were rinsed with PBST (PBS containing 0.5% Triton X-100) and incubated with secondary antibodies (biotin-labelled goat anti-rabbit, 1:300, A0277, Beyotime) for 2 h at room temperature. Subsequently, the sections were washed again and incubated with enzyme-labelled streptavidin (1:300, Beyotime) for 1.5 h. The visual signal was developed using a DAB kit (ZLI-9017, ZSGB Biotech, Beijing, China) and counterstained with haematoxylin. Post-staining procedures, including dehydration and mounting with neutral resin, mirrored those described for H&E staining. Representative images were captured from five randomly selected fields for each section using an Olympus microscope (BX51, Olympus, Tokyo, Japan), and quantitative analysis of the immunohistochemical signals was performed using ImageJ software.

### 2.7. Determination of Antioxidant Enzyme Activities and Oxidative Stress Markers

Spleen tissues were homogenised and centrifuged at 4 °C (12,000× *g*, 10 min) to obtain clear lysates. Protein concentrations were determined using a protein assay kit (CW0014, CWBIO, Taizhou, China). Following the manufacturer’s instructions, the levels of malondialdehyde (MDA, S0131S), total antioxidant capacity (T-AOC, S0116, Beyotime), glutathione peroxidase (GSH-px, S0056, Beyotime), superoxide dismutase (SOD, S0101S, Beyotime), and catalase (CAT, S0051, Beyotime) were measured using specific commercial kits.

### 2.8. RNA Extraction and Quantitative Real-Time PCR (RT-qPCR)

Total RNA was extracted from spleen tissues using TRIzol reagent (R401-01-AA, Vazyme, Nanjing, China). Quantitative reverse transcription polymerase chain reaction was performed using the Applied Biosystems StepOnePlus RT-PCR System (4376600, Applied Biosystems, Thermo Fisher, Waltham, MA, USA). The final reaction volume was 20 μL, comprising 10 μL SYBR Green PCR Master Mix (Q121-02, Vazyme, Nanjing, China), 2 μL cDNA, 0.4 μL forward primer, 0.4 μL reverse primer, and 7.2 μL DEPC-treated water. Relative mRNA expression levels were quantified using the 2^(−ΔΔCt)^ method. Target genes for RT-qPCR were selected a priori based on the literature and our study hypothesis, focusing on inflammatory mediators [40], oxidative-stress responses [41], apoptosis-related markers [42], and glucocorticoid receptor signalling [43]. RNA-seq was performed subsequently as an independent transcriptome-wide analysis to determine whether the broader gene-expression profile was consistent with these pre-specified targets. The specific primer sequences used for these selected genes are detailed in Supplementary Table S2.

### 2.9. Enzyme-Linked Immunosorbent Assay (ELISA)

Spleen tissue homogenates were processed, with protein concentration determined using the BCA Protein Assay Kit (CW0014S, CWBIO). The levels of inflammatory cytokines, including IL-6, IL-1β, TNF-α, IL-10, and IL-5 (SL09000, SL09152, SL08798, SL09012, SL08700, SLCY, Beijing, China), as well as oxidative stress markers and antioxidant enzyme activities, such as MDA, CAT, GSH-px, and T-AOC, were quantified using specific ELISA kits (SL09388, SL31682, SL15148, SL08730, SLCY). All procedures were performed in strict accordance with the manufacturer’s instructions. All biochemical parameters were normalised to the total protein concentration of the respective samples. The intra-assay and inter-assay coefficients of variation for these kits were less than 9% and 11%, respectively.

### 2.10. Primary Splenic Cell Extraction and Culture

After removal, the mouse spleen was placed in D-Hank’s solution (BL559A, Biosharp, Beijing, China) and ground through a 70-mesh cell sieve to obtain a single-cell suspension. The suspension was layered onto the mouse lymphocyte separation medium and centrifuged at 800× *g* for 20 min at room temperature. The mononuclear cell layer at the interface was collected and washed twice with PBS. Cell viability was confirmed using Trypan blue staining. The isolated splenocytes were then resuspended in RPMI Medium 1640 containing 10% FBS (10270-106, Gibco, Waltham, MA, USA) and 1% penicillin-streptomycin at a density of 2.5 × 10^6^ cells/mL for subsequent experimental analysis.

For all in vitro assays, including the ConA/LPS stimulation and pharmacological treatments, primary splenocytes were derived from independent biological replicates (n = 5 per group for CCK-8 assays; n = 3 per group for Western blot analysis), and each sample was analysed with three technical replicates (triplicate wells).

### 2.11. Cell Viability Assay (CCK-8)

To evaluate the protective effects of RU486 against CORT-induced suppression, primary splenocytes seeded in 96-well plates were stimulated with ConA (5 μg/mL) to induce T-cell proliferation or LPS (10 μg/mL) to induce B-cell proliferation. Subsequently, CORT (final concentration 2 μM; HY-B1618, MCE) and RU486 (final concentration 10 μM; HY-13683, MCE) were added to the culture media. The plates were incubated in a CO_2_ incubator (5% CO_2_, 37 °C) for 44 h. Then, 10 μL of CCK-8 reagent was added to each well, followed by an additional 2 h incubation. The optical density (OD) at 450 nm was measured using a microplate reader to assess cell viability according to the manufacturer’s protocol.

### 2.12. Protein Extraction and Western Blotting

Proteins from spleen tissue or cells were lysed using RIPA lysis buffer (CW2333S, CWBIO) supplemented with 1% protease inhibitor (CW2200S, CWBIO) and 1% phosphatase inhibitor (CW2383S, CWBIO), followed by centrifugation at 12,000× *g* for 15 min at 4 °C. Protein concentration in the supernatant was determined using a protein assay kit (CW0014, CWBIO). Based on the protein concentrations obtained, all samples were adjusted to a final concentration of 2 μg/μL using the remaining protein lysate and 5× loading buffer, then heated at 100 °C for 10 min to ensure complete protein denaturation.

Equal amounts of protein (30 μg per lane) were separated by 10% sodium dodecyl sulphate-polyacrylamide gel electrophoresis (SDS-PAGE) and subsequently transferred onto polyvinylidene fluoride (PVDF) membranes (Millipore, Billerica, MA, USA). PVDF membranes were blocked with 5% skimmed milk in TBST for 1.5 h at room temperature. The membranes were then incubated with specific primary antibodies at 4 °C overnight. After washing three times with TBST, the membranes were incubated with horseradish peroxidase-conjugated goat anti-rabbit IgG (1:8000; CW0103, CWBIO) or goat anti-mouse IgG (1:8000; CW0102, CWBIO) for 90 min at room temperature. Visualisation was performed using an automated digital gel imaging system (5200, Tanon, Shanghai, China). Densitometric analysis was performed using ImageJ software (Scion Corp., Frederick, MD, USA). Relative protein levels were expressed as a percentage of the control group, with data normalised to the density of GAPDH.

Primary antibodies: Primary antibodies included rabbit anti-PCNA (10205-2-AP, 1:1000, Proteintech), rabbit anti-GR (24050-1-AP, 1:2000, Proteintech), rabbit anti-NF-κB p-p65 (82335-1-RR, 1:1000, Proteintech), rabbit anti-Nrf2 (16396-1-AP, 1:1000, Proteintech), rabbit anti-HO-1 (10701-1-AP, 1:1000, Proteintech), and mouse anti-GAPDH (60004-1-IG, 1:8000, Proteintech).

### 2.13. Spleen Transcriptomics Sequencing Workflow

#### 2.13.1. Extraction of RNA from Spleen Tissue

Total RNA was extracted from mouse spleen tissues using TRIzol reagent (R401-01-AA, Vazyme). Subsequently, mRNA was enriched for library construction. Three animals were selected per group in the LD, dBL, and dBL + Metyrapone groups. Nucleic acid samples underwent concentration measurement and purity parameter analysis via spectrophotometric techniques, alongside standardised integrity assessment using a commercial RNA quality detection system.

#### 2.13.2. Transcriptome Sequencing Library Preparation

The RNA input for each sample was set at 1 μg. Library construction was performed using the Hieff NGS Ultima Dual-Mode mRNA Library Preparation Kit (Yeasen, Shanghai, China), with unique molecular identifiers (UMIs). The primary workflow is as follows: Initially, poly(A) RNA enrichment was achieved using oligo(dT)-modified magnetic carriers. Following first-strand complementary DNA synthesis, second-strand synthesis commenced immediately. A dual-enzyme system converted cDNA ends to blunt-end structures. This was followed by 3′-end adenylation and ligation with NEBNext hairpin adapters. Nucleic acid purification was performed using AMPure XP magnetic beads (Beckman Coulter, Brea, CA, USA) . Following the addition of 3 μL USER enzyme (New England Biolabs, Ipswich, MA, USA), a dual-temperature treatment was executed: incubation at 37 °C for 15 min, followed by 95 °C for 5 min. The final stage involves exponential amplification using a high-precision polymerase with universal amplification primers and specific labelling primers. The final product underwent magnetic bead-based enrichment, followed by library integrity assessment and quantitative analysis using the Agilent 2100 Bioanalyzer (Agilent Technologies, Santa Clara, CA, USA). Sequencing analysis employed the Illumina NovaSeq 6000 platform (Illumina, San Diego, CA, USA), performing 150 bp paired-end sequencing according to standard operating procedures. This yielded high-quality paired-end reads (Q30 ≥ 90%).

#### 2.13.3. Data Analysis

Following preprocessing of raw sequencing data, multidimensional analysis was conducted using standardised modules integrated within the BMK Cloud platform (www.biocloud.net, accessed on 4 September 2025). The specific workflow comprised: (1) Quality control screening of raw sequences to obtain high-quality reads; (2) Sequence mapping via genome-wide alignment algorithms; (3) Multiparametric assessment of library construction quality; (4) Transcriptomic structural feature analysis; (5) Quantitative comparison of differentially expressed gene profiles; (6) In-depth analysis, including gene ontology annotation and KEGG metabolic pathway enrichment analysis.

### 2.14. Statistical Analysis

No animals were excluded from the study after group allocation. The biological replicate was defined as an individual animal, and technical replicates were not treated as independent biological replicates. Sample sizes were selected on the basis of previous studies using comparable light-exposure and splenic immune endpoints, practical feasibility, and the 3Rs principle. A formal a priori power calculation was not performed. Five biological replicates per group were used for morphological, molecular, and cellular functional analyses. Three biological replicates per group were used for cell protein-related Western blot analyses and transcriptomic sequencing; these analyses were interpreted as supportive rather than standalone definitive evidence. The effective biological sample size for each assay is reported in the corresponding figure legends.

All data were statistically analysed using GraphPad Prism (version 10.0; GraphPad Software, San Diego, CA, USA) and are presented as mean ± standard error of the mean (SEM). Differences among multiple experimental groups were assessed using a one-way analysis of variance (ANOVA) followed by Tukey’s post hoc test. For these physiological, histological, biochemical, qPCR, cellular functional, and protein-expression analyses, differences were considered statistically significant at *p* < 0.05. For the transcriptomic analysis, multiple-testing correction was applied to control the false discovery rate (FDR). Genes meeting the thresholds of FDR < 0.01, *p* < 0.01, and fold change (FC) > 2 were considered differentially expressed.

## 3. Results

### 3.1. Nocturnal dBL Exposure Is Associated with Splenic Histopathological Changes and Reduced Cellular Proliferation

**Figure 2:**
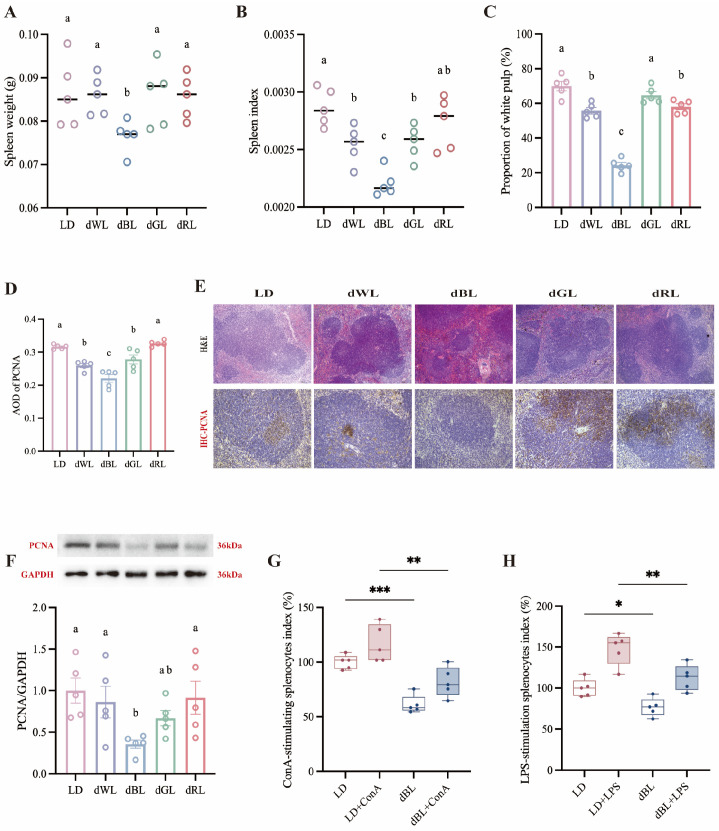
**Effects of dLAN on splenic immune function in mice.** (**A**) Spleen weight and (**B**) spleen index in mice under different nocturnal light conditions. (**C**,**D**) Quantitative analyses of the (**C**) white pulp area (%) and (**D**) AOD values for PCNA-positive cells, respectively. (**E**) Representative images of splenic haematoxylin and eosin (H&E) staining (scale bar = 100 μm) and PCNA immunohistochemical staining (scale bar = 50 μm). (**F**) Representative Western blot bands and densitometric analyses of PCNA protein levels. (**G**,**H**) Stimulation indices (%) of ConA- and LPS-stimulated splenocytes. Data are presented as mean ± SEM (*n* = 5) and were analysed via one-way ANOVA. For (**G**,**H**), Tukey’s post hoc test was employed for multiple comparisons, and statistical significance is indicated by asterisks (* *p* < 0.05, ** *p* < 0.01, *** *p* < 0.001). For all other panels, different letters indicate significant differences among groups (*p* < 0.05), while exact *p*-values are provided in the corresponding Results section of the text. LD: control group exposed to the standard light–dark cycle group; dWL: dim white light group; dBL: dim blue light group; dGL: dim green light group; dRL: dim red light group.

Statistical analysis indicated that relative to the control (LD), spleen weights remained largely unaffected under broad-spectrum or long-wavelength exposures, with non-significant reductions ranging from 0.39% to 0.79% in the dim white light (dWL) group, dim green light (dGL) group, and dim red light (dRL) group (*p* = 0.8573–0.9284). Among all spectra, only the dim blue light (dBL) cohort showed a significant decline, reaching 11.26% (*p* = 0.0170) (Figure 2A). This trend was corroborated by the spleen index, which revealed a reduction of 23.02% in the dBL group relative to the LD control (*p* < 0.0001). While decreases were also observed in the dWL and dGL cohorts, with reductions of 11.29% (*p* = 0.0060) and 10.56% (*p* = 0.0094), respectively, the dBL group presented the most severe reduction. In contrast, dRL exposure was associated with a slight, non-significant reduction of 4.83% (*p* = 0.2033) (Figure 2B).

Prompted by these preliminary observations, histopathological examinations using H&E staining were performed to evaluate structural alterations. The results demonstrated that nocturnal light exposure was associated with spectrum-dependent differences in splenic microarchitecture. Quantitative analysis revealed reductions in the proportion of the white pulp area by 20.35% and 17.06% under dWL and dRL conditions, respectively (*p* < 0.0001; *p* = 0.0050), alongside a non-significant decrease of 7.52% in the dGL group (*p* = 0.0849). Among all experimental cohorts, dBL exposure was associated with the greatest reduction in the white pulp area, reaching 65.27% relative to the LD (*p* < 0.0001) (Figure 2C,E). This morphological alteration indicates disruption to the integrity of the spleen’s immune functional zones.

To further assess splenic proliferative status, PCNA expression was examined. Proliferating cell nuclear antigen (PCNA) protein levels in the control and other spectral groups were 41.90–80.57% higher than those of the dBL group (*p* = 0.0059–0.0148), while the dGL cohort showed a non-significant 87.53% increase relative to the dBL group (*p* = 0.1515) (Figure 2F). Immunohistochemical (IHC) staining further confirmed this in situ, demonstrating a significant reduction in PCNA-positive cells within the white pulp of the dBL-exposed group, accompanied by a 17.77–43.41% decrease in signal intensity relative to the other experimental cohorts (*p* = 0.0001–0.0048) (Figure 2D,E). Consistent with these proliferation-related changes, apoptosis-related transcripts showed corresponding alterations in the dBL group, as shown by increased Bax and Caspase-3 expression and reduced Bcl2 expression (Figure S1A–C).

Given the observed changes in splenic morphology and proliferation-related markersin vitro activation and proliferation assays were conducted on primary splenocytes. The results confirmed that cells from dBL-exposed mice demonstrated suppressed activation responses, with reductions of 42.78% (*p* = 0.0070) and 31.10% (*p* = 0.0137) relative to the LD control following ConA and LPS stimulation, respectively (Figure 2G,H). Collectively, these findings indicate that, under an HFD background, nocturnal dBL exposure was associated with altered splenic tissue architecture, reduced proliferation-related markers, and impaired activation responses in splenic cells.

### 3.2. Nocturnal dBL Exposure Is Associated with Splenic Oxidative Stress and a Pro-Inflammatory Microenvironment

**Figure 3:**
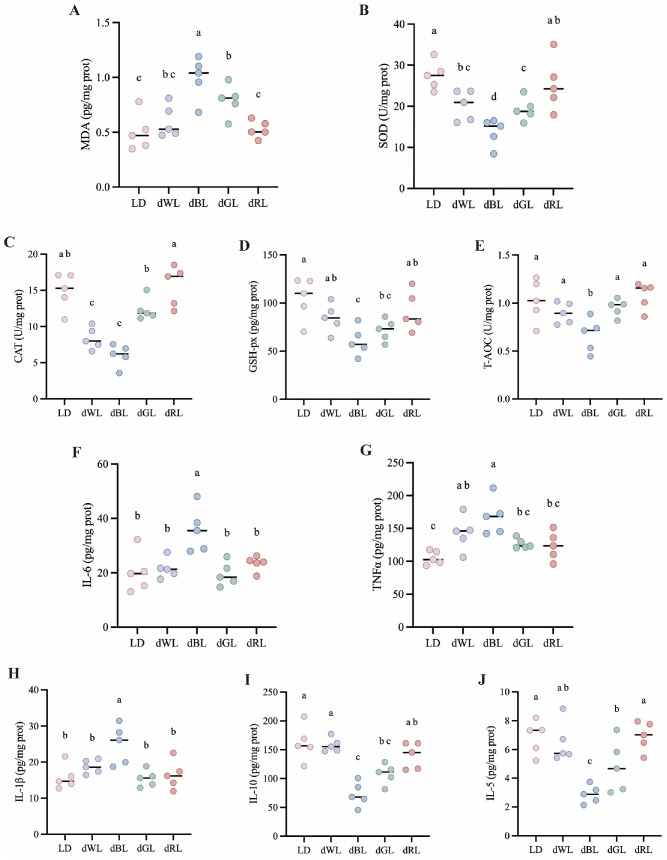
**Nocturnal dBL-associated splenic oxidative stress and inflammatory responses.** (**A**–**E**) Levels or activities of MDA, SOD, CAT, GSH-px, and T-AOC in the spleen. (**F**–**J**) Protein levels of IL-6, TNF-α, IL-1β, IL-10 and IL-5 in the spleen. Data are presented as mean ± SEM (*n* = 5). Statistical significance was determined as described in Figure 2.

To examine whether wavelength-specific nocturnal light exposure was associated with altered splenic redox status, redox-related indices were compared across the light-exposure groups. Among the monochromatic light exposure conditions, the dBL cohort showed the most distinct oxidative profile, with malondialdehyde (MDA) levels increasing by 20.48–49.67% relative to the LD control and other monochromatic groups (*p* = 0.001–0.0472) (Figure 3A).

Antioxidant indices displayed the opposite pattern. Superoxide dismutase (SOD) activities in dBL-exposed spleens were 40.11–99.26% lower than those in the LD control and other cohorts (*p* = 0.0001–0.0226). Catalase (CAT) levels in the LD and other monochromatic groups were 4.95–59.41% higher than those of the dBL group (*p* < 0.0001), while the dWL group exhibited a non-significant elevation of 38.89% over the dBL baseline (*p* = 0.0877). Similarly, GSH-px activities in the dBL group declined by 40.15–73.59% relative to the LD control and other groups (*p* = 0.0006–0.0381), whereas the dGL group exhibited a non-significant 18.96% elevation relative to the dBL group (*p* = 0.3069). These changes were accompanied by reduced local total antioxidant capacity (T-AOC) in dBL-exposed spleens, with reductions of 35.02–54.49% relative to other cohorts (*p* = 0.0004–0.0280) (Figure 3B–E). Together, these redox-related changes indicate that, under the HFD background, dBL exposure was associated with a more pronounced disturbance of splenic oxidative balance than the other spectra tested.

Inflammatory cytokine profiles showed a similar wavelength-related pattern. Compared with the LD control and other spectral cohorts, IL-6 protein levels in the dBL group increased by 34.52–45.44% (*p* = 0.0018–0.0205). This spectral specificity was further evidenced by TNF-α, with protein levels in the dBL group increasingreached 24.50–37.29% for protein levels (*p* = 0.0001–0.0047). In contrast, the dWL group exhibited only a non-significant elevation of 15.05% for TNF-α protein (*p* = 0.0651) over the dBL levels. IL-1β protein levels also increased in the dBL cohort by 24.70–37.99% (*p* = 0.0008–0.0146), whereas the other monochromatic groups remained near baseline levels (Figure 3F–H). The corresponding *Il6*, *Tnf*, and *Il1b* mRNA profiles followed the same overall direction (Figure S1D–F).

By contrast, anti-inflammatory and immunomodulatory cytokines were lower in dBL-exposed spleens. IL-10 protein levels in the LD and most other cohorts were 92.25–122.26% higher compared with the dBL group (*p* = 0.0001–0.0125), whereas the dGL group showed a non-significant elevation of 48.26% over the dBL baseline (*p* = 0.1182). Consistent with this suppressive trend, IL-5 protein expression was significantly lower in the dBL cohort, with other groups demonstrating levels 67.25–140.25% higher than the dBL baseline (*p* = 0.0001–0.0256) (Figure 3I,J). The corresponding *Il10* and *Il5* mRNA profiles showed a similar direction (Figure S1G,H).

Together, these findings suggest that nocturnal dBL exposure is associated with disturbed splenic redox balance and a cytokine profile shifted towards a pro-inflammatory microenvironment under the HFD background.

### 3.3. Nocturnal dBL Exposure Is Accompanied by Altered CORT-GR Signalling and Reciprocal Changes in Inflammatory and Antioxidant Pathways

**Figure 4:**
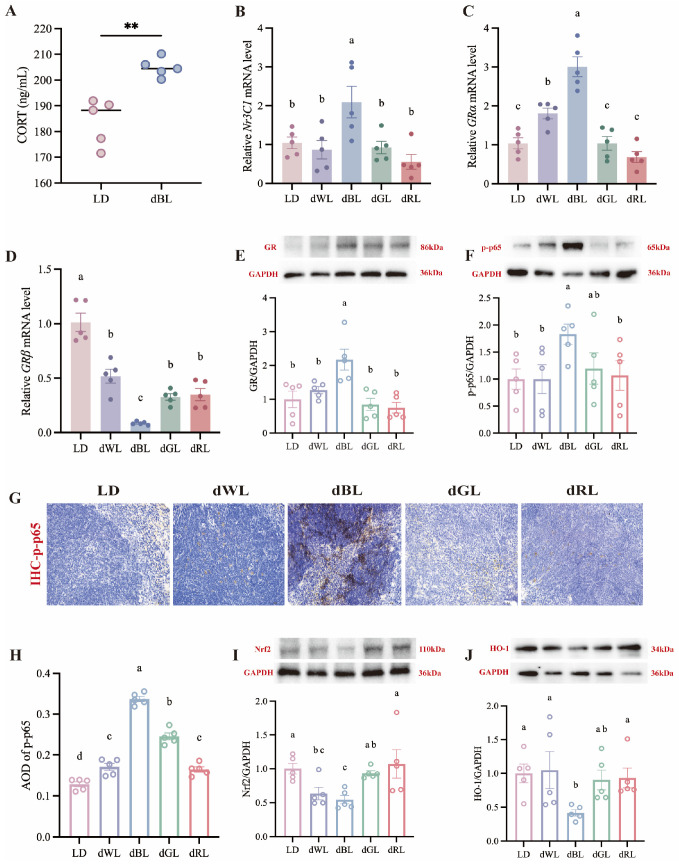
**Nocturnal dim blue light is associated with altered CORT-GR signalling and reciprocal changes in inflammatory and antioxidant pathways.** (**A**) Plasma CORT levels. (**B**–**D**) Relative mRNA levels of Nr3c1, GRα, and GRβ. (**E**) Representative Western blot bands and densitometric analyses of GR protein levels. (**F**) Representative Western blot bands and densitometric analyses of p-p65 protein levels. (**G**,**H**) Representative p-p65 immunohistochemical staining images (scale bar = 50 μm) and corresponding AOD values. (**I**,**J**) Representative Western blot bands and densitometric analyses of Nrf2 and HO-1 protein levels. Data are presented as mean ± SEM (*n* = 5). Statistical significance was determined as described in Figure 2.

Following the observation of splenic tissue degeneration, we examined whether nocturnal dBL exposure was associated with changes in systemic corticosterone secretion and splenic GR signalling. ELISA results revealed that, compared with the LD control, nocturnal dBL exposure was associated with a 10.03% increase in serum corticosterone (CORT) levels (*p* = 0.0013) (Figure 4A). To assess the splenic response to this endocrine signal, GR expression was examined. qPCR analysis identified a significant upregulation of Nr3c1 and its primary functional isoform GRα (Figure 4B,C). Consistent with these transcriptional changes, Western blot analysis detected a 41.33–65.46% increase in total GR protein levels within the dBL group (*p* = 0.0001–0.0071) (Figure 4E). Notably, while the functional alpha isoform was elevated, the mRNA expression of the inhibitory isoform *GRβ* was significantly suppressed following dBL exposure, with transcript levels in the other cohorts being 183.93–989.07% higher than those of the dBL group (*p* = 0.0001–0.0383) (Figure 4D).

Building on theevidence of altered GR signalling, the downstream inflammatory pathway, nuclear factor kappa-light-chain-enhancer of activated B cells (NF-κB), was further assessed, with increased Nfkb1 mRNA expression observed in the dBL group (Figure S1I). Western blot results indicated that the dBL group displayed a 41.65–45.39% increase in p65 phosphorylation (p-p65) compared with other groups (*p* = 0.0266–0.0400), whereas the dGL group showed a non-significant 34.69% elevation (*p* = 0.0823) (Figure 4F). Immunohistochemical staining revealed more intense p-p65 immunoreactivity in dBL-exposed spleens, with AOD values in the other experimental groups being 27.22–61.91% lower than those in the dBL group (*p* = 0.001–0.0050) (Figure 4G,H). Together, these findings indicate that nocturnal dBL exposure was accompanied by increased splenic NF-κB-related inflammatory signalling.

Beyond inflammatory signalling, the key antioxidant response pathway, nuclear factor erythroid 2-related factor 2 (Nrf2)/haem oxygenase-1 (HO-1), was evaluated to examine the redox-related alterations. Reduced *Nfe2l2* and *Hmox1* mRNA expression in the dBL group provided transcript-level context for the protein analyses (Figure S1J,K). Nrf2 abundance in most other groups were 71.65–96.60% higher than the dBL baseline (*p* = 0.0036–0.0238), with the dWL group exhibiting a non-significant elevation of 16.49% over the dBL baseline (*p* = 0.5798) (Figure 4I). In parallel, HO-1 protein levels in most other cohorts were 139.46–150.75% higher than the dBL baseline (*p* = 0.0146–0.0413), whereas the dGL group showed a non-significant elevation of 115.94% over the dBL baseline (*p* = 0.0530) (Figure 4J). These findings suggest that dBL-associated splenic alterations were accompanied by reduced Nrf2/HO-1-associated antioxidant responses.

In summary, nocturnal dBL exposure is accompanied by elevated circulating CORT, altered splenic GR expression, enhanced NF-κB signalling, and suppressed Nrf2/HO-1 antioxidant responses. Together, these concomitant changes are consistent with the involvement of CORT-GR signalling as a contributory pathway associated with dBL-related splenic immune alterations.

### 3.4. Pharmacological Modulation of CORT-GR Signalling Partially Attenuates dBL-Associated Splenic Alterations

**Figure 5:**
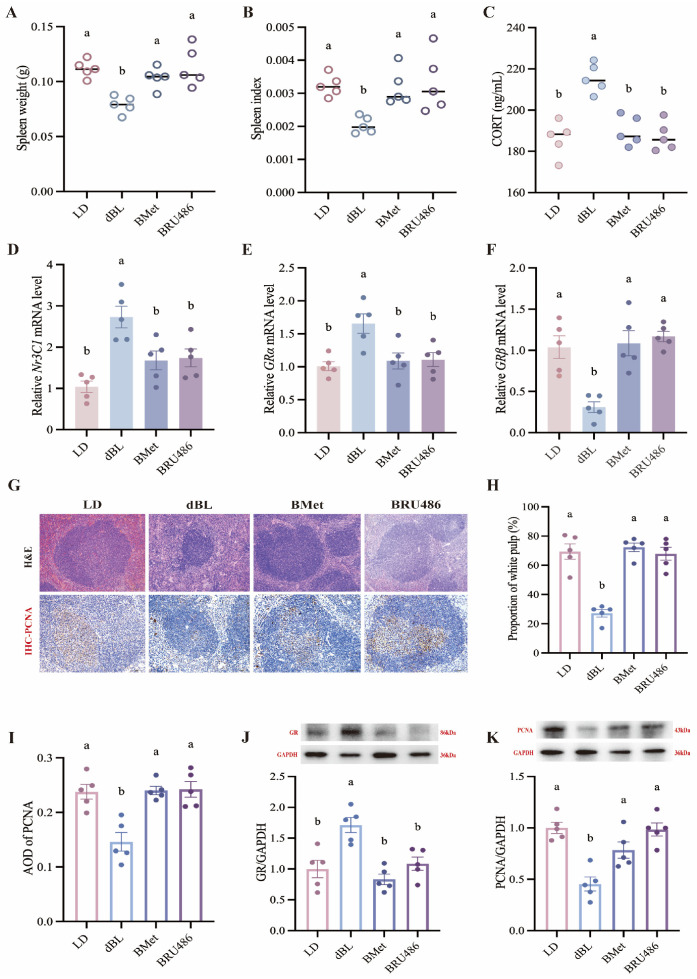
**Effects of pharmacological intervention on dBL-associatedspleen morphology andGR expression.** (**A**) Spleen weight in mice following administration of Metyrapone and RU486. (**B**) Spleen index in mice. (**C**) Plasma CORT levels. (**D**–**F**) Relative mRNA levels of Nr3c1, GRα, and GRβ in the spleen. (**G**) Representative images of splenic haematoxylin and eosin (H&E) staining (scale bar = 100 μm) and PCNA immunohistochemical staining (scale bar = 50 μm). (**H**) Quantitative analysis of the white pulp area (%). (**I**) AOD values of PCNA immunohistochemical staining. (**J**) Representative Western blot bands and densitometric analyses of GR protein levels. (**K**) Representative Western blot bands and densitometric analyses of PCNA protein levels. Data are presented as mean ± SEM (*n* = 5). Statistical significance was determined as described in Figure 2. LD: control group exposed to the standard light–dark cycle; dBL: dim blue light group; BMet: dim blue light with Metyrapone group; BRU486: dim blue light with RU486 group.

To examine the contribution of CORT-GR signalling to the observed splenic phenotype, in vivo pharmacological intervention experiments were conducted using an independent pharmacological cohort. This cohort was subjected to the same 12-week lighting conditions but received additional treatment with the CORT synthesis inhibitor Metyrapone (BMet) or the GR antagonist RU486 (BRU486) during weeks 11–12. Spleen-weight analysis indicated that pharmacological modulation of CORT-GR signalling partially attenuated the dBL-associated reduction in spleen weight, with BMet and BRU486 groups showing gains of 30.99% and 43.68%, respectively, compared with the dBL cohort (*p* = 0.0190; *p* = 0.0012) (Figure 5A). Correspondingly, both interventions attenuated the dBL-associated reductions in spleen index, achieving gains of 55.53% and 62.39% in the BMet and BRU486 groups, respectively (*p* = 0.0269; *p* = 0.0122) (Figure 5B). These findings indicate that modulation of this axis partially attenuates splenic alterations under this specific lighting condition.

The pharmacodynamic effects of these interventions were then assessed to further examine the involvement of CORT-GR signalling in the observed splenic changes.

 ELISA assays indicated that Metyrapone (BMet) and RU486 (BRU486) reduced serum CORT towards basal levels, representing significant decreases of 11.86% and 13.12% (*p* = 0.0003; *p* < 0.001) (Figure 5C). In parallel, Western blot results showed that both BMet and BRU486 attenuated the elevated expression of GR protein in the spleen, which declined by 51.30% and 36.51%, respectively (*p* = 0.0003; *p* = 0.0073) (Figure 5J). At the transcriptional level, the dBL-associated upregulations of the Nr3c1 gene and its functional isoform GRα were significantly attenuated upon pharmacological treatment (Figure 5D,E). Notably, GRβ expression increased, exhibiting increases of 249.95% and 276.68% relative to the dBL group (*p* = 0.0008; *p* = 0.0003) (Figure 5F). Together, these results suggest that pharmacological modulation shifted CORT-GR-related alterations towards baseline. providing an in vivo framework to further investigate the functional shift towards baseline of the dBL-challenged spleen.

Complementing these systemic improvements, histological examination via H&E staining showed partial preservation of splenic architecture and white pulp area, representing increases of 166.07% and 149.90%, respectively (both *p* < 0.0001) (Figure 5G,H). Immunohistochemical analysis further substantiated a substantial improved in the proportion of PCNA-positive cells within the splenic germinal centres, with increases of 64.49% and 65.88% in the intervention groups (*p* = 0.0008; *p* = 0.0007) (Figure 5G,I). In alignment with the Western blot results, biochemical assays evidenced that both BMet and BRU486 significantly upregulated PCNA protein expression levels in the spleen by 72.95% and 117.07%, respectively (*p* = 0.0142; *p* = 0.0002) (Figure 5K), providing quantitative protein evidence for the in situ increased of proliferative activity within the germinal centres.

**Figure 6:**
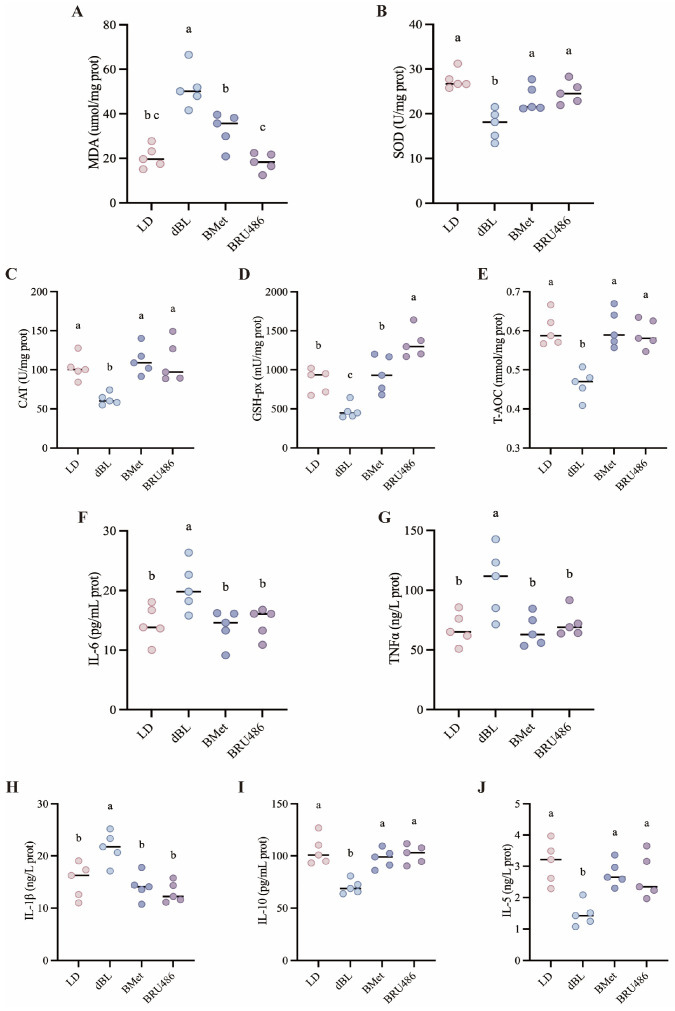
**Alterations in dBL-associated splenic oxidative stress and inflammatory responses following pharmacological intervention.** (**A**–**E**) Levels or activities of MDA, SOD, CAT, GSH-px, and T-AOC in the spleen. (**F**–**J**) Protein levels of IL-6, TNF-α, IL-1β, IL-10 and IL-5 in the spleen. Data are presented as mean ± SEM (*n* = 5). Statistical significance was determined as described in Figure 2.

Alongside the structural changes described above, pharmacological intervention was associated with partial improvement of the splenic redox microenvironment. BMet and BRU486 reduced the accumulation of the lipid peroxidation product MDA, reducing its levels by 36.37% and 64.50% relative to the dBL cohort, respectively (*p* = 0.0023; *p* < 0.0001). In parallel, antioxidant enzyme activities increased following pharmacological intervention. SOD levels improved by 33.14% and 40.40% (*p* = 0.0197; *p* = 0.0045), while CAT activities increased by 79.29% and 76.39% (*p* = 0.0030; *p* = 0.0042) in the BMet and BRU486 groups, respectively. These enzymatic improvements were further mirrored by GSH-px and T-AOC levels. GSH-px activities rose by 100.42% and 182.68% (*p* = 0.0029; *p* < 0.0001), while T-AOC levels increased by 27.75% and 30.60% (*p* = 0.0003; *p* = 0.0007) following Metyrapone and RU486 treatment, relative to the dBL baseline (Figure 6A–E). Notably, these specific antioxidant capacities were not only improved but also reached levels exceeding the baseline observed in the LD control.

Pharmacological intervention was also associated with attenuation of the splenic pro-inflammatory profile. Treatment with Metyrapone and RU486 was associated with lower IL-6 protein levels, with decreases of 32.51% and 28.87% (*p* = 0.0208; *p* = 0.0432), respectively, relative to the dBL group. Similarly, TNF-α protein concentrations were significantly reduced by 37.90% and 32.47% (*p* = 0.0130; *p* = 0.0358). IL-1β protein levels decreased by 34.73% and 39.82% (*p* = 0.0029; *p* = 0.0008) in the BMet and BRU486 groups, respectively (Figure 6F–H). The corresponding *Il6*, *Tnf*, and *Il1b* mRNA profiles followed the same overall direction (Figure S2A–C).

Correspondingly, anti-inflammatory mediators increased following pharmacological intervention. IL-10 protein levels increased by 39.00% and 44.54% in the BMet and BRU486 cohorts, respectively, compared with the dBL baseline (*p* = 0.0026; *p* = 0.0008). IL-5 concentrations rose by 88.49% and 81.63% following pharmacological intervention (*p* = 0.0102; *p* = 0.0180) (Figure 6I,J). These protein-level changes were paralleled by corresponding alterations in *Il10* and *Il5* mRNA expression (Figure S2D,E). Together, these data indicate that CORT-GR modulation was accompanied by a shift towards a less pro-inflammatory and more immunoregulatory splenic cytokine profile.

**Figure 7:**
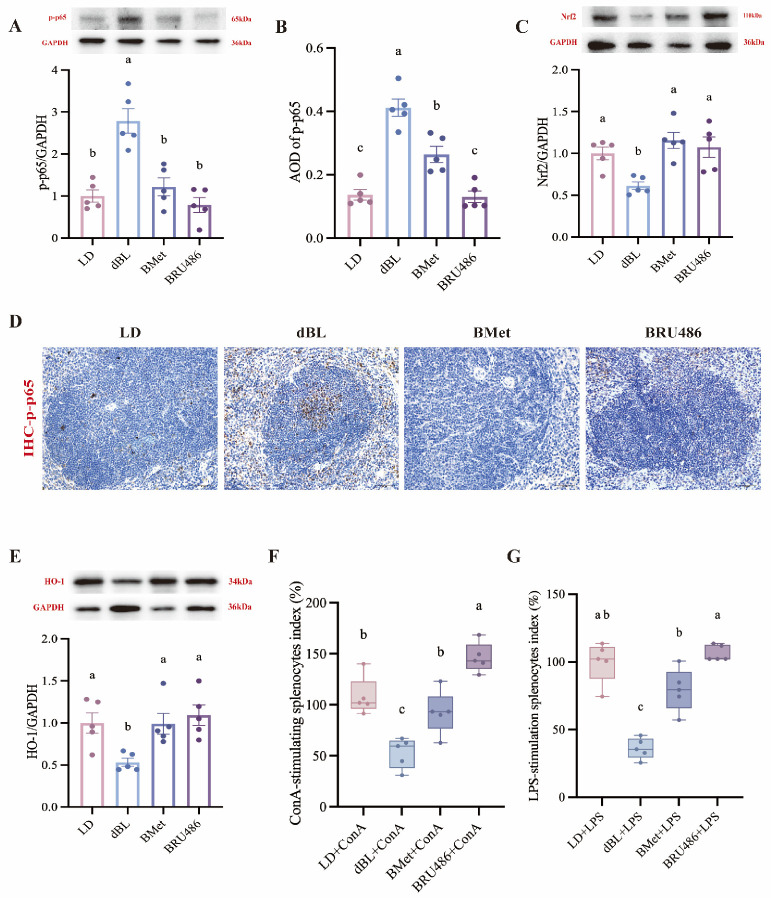
**Modulation of dBL-associated splenic molecular pathways and primary splenocyte function by pharmacological intervention.** (**A**) Representative Western blot bands and densitometric analyses of p-p65 protein levels. (**B**) AOD values of p-p65 immunohistochemical staining. (**C**) Representative Western blot bands and densitometric analyses of Nrf2 protein levels. (**D**) Representative images of p-p65 immunohistochemical staining (scale bar = 50 μm). (**E**) Representative Western blot bands and densitometric analyses of HO-1 protein levels. (**F**,**G**) Stimulation indices (%) of ConA- and LPS-stimulated splenocytes. Data are presented as mean ± SEM (*n* = 5). Statistical significance was determined as described in Figure 2.

To further examine pathway-level changes associated with the redox-inflammatory alterations described above, NF-κB and Nrf2/HO-1 signalling were assessed in the pharmacological cohort. The dBL-associated increase in Nfkb1 (NF-κB) mRNA expression was attenuated following BMet and RU486 treatment (Figure S2F). Western blot analysis indicated that p-p65 protein levels decreased by 56.27% and 71.72% in the BMet and BRU486 groups, respectively, compared with the dBL cohort (*p* = 0.0004; *p* < 0.0001) (Figure 7A). Immunohistochemical analysis revealed a lower p-p65 signal in the intervention groups, with AOD values reduced by 35.78% and 68.34%, respectively (*p* = 0.0013; *p* < 0.0001) (Figure 7B,D). The combined protein and histological findings suggest attenuation of dBL-associated NF-κB-related inflammatory signalling following pharmacological intervention.

The Nrf2/HO-1 antioxidant pathway showed an opposite pattern. *Nfe2l2* (Nrf2) and *Hmox1* (HO-1) mRNA expression increased relative to the dBL group following pharmacological intervention (Figure S2G,H). Consistent with this transcriptional pattern, Nrf2 protein levels rose by 88.55% and 75.22% (*p* = 0.0026; *p* = 0.0099) and HO-1 by 85.84% and 105.17% (*p* = 0.0401; *p* = 0.0105) in the BMet and BRU486 groups, respectively (Figure 7C,E). Together, these changes suggest enhanced Nrf2/HO-1-related antioxidant responses after CORT-GR modulation, with apoptosis-related transcripts showing a directionally consistent shift away from a pro-apoptotic profile (Figure S2I–K).

In response to mitogen stimulation, splenic proliferative responses were higher following pharmacological intervention. Following LPS stimulation, proliferative activity increased by 119.52% and 194.59% in the BMet and BRU486 groups, respectively, compared with the dBL-exposed cohort (*p* = 0.0002; *p* < 0.0001). Similarly, ConA-induced proliferation rose by 74.50% and 175.70% in the corresponding treatment cohorts (*p* = 0.0124; *p* < 0.0001) (Figure 7F,G).

### 3.5. In Vitro Evidence That CORT Is Associated with Suppressed Primary Splenocyte Activation Responses Involving GR-Dependent Signalling

**Figure 8:**
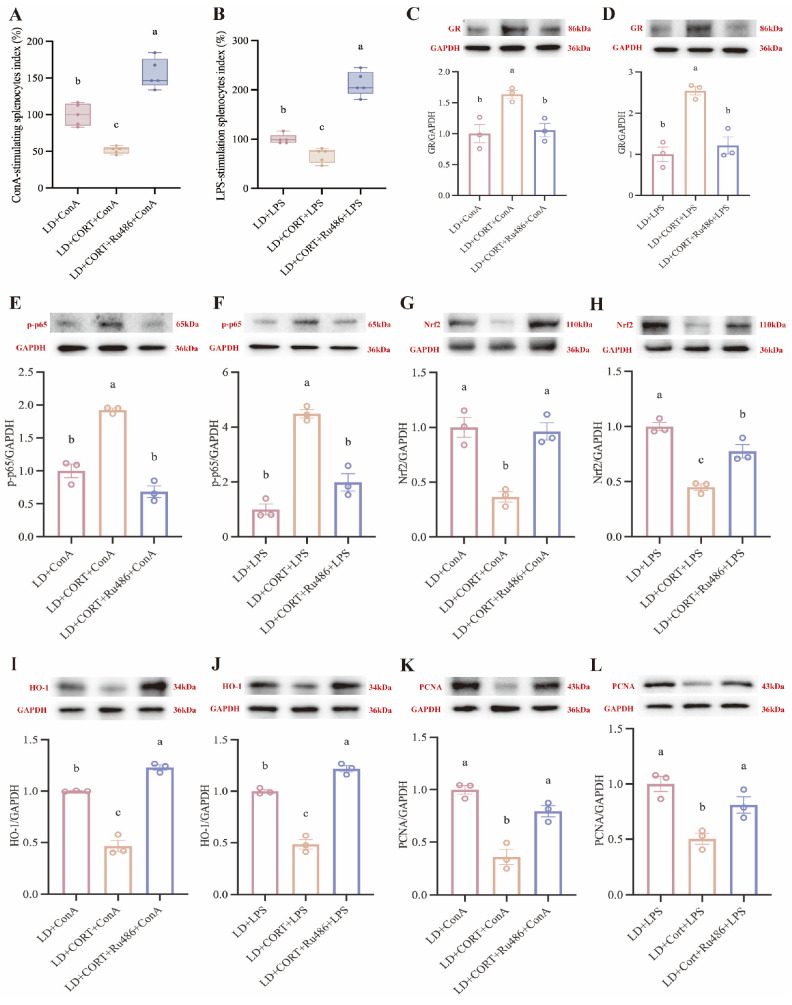
**Effects of CORT and RU486 on the activation and intracellular signalling of primary splenocytes in vitro.** Primary splenocytes were isolated and stimulated with ConA or LPS to assess T-cell- and B-cell-driven responses, respectively. (**A**,**B**) Stimulation indices (%) of ConA- and LPS-stimulated splenocytes (*n* = 5). (**C**–**L**) Representative Western blot bands and corresponding densitometric analyses of GR (**C**,**D**), p-p65 (**E**,**F**), Nrf2 (**G**,**H**), HO-1 (**I**,**J**), and PCNA (**K**,**L**) protein levels in the respective mitogen-stimulated splenocyte populations. Data are presented as mean ± SEM (**C**–**L**: *n* = 3). Statistical significance was determined as described in Figure 2.

To examine primary splenocyte responses under controlled conditions, an in vitro culture model using primary splenocytes was established. Experimental data showed that CORT treatment significantly attenuated the activation capacity of primary splenocytes, leading to a decrease in the stimulation index of ConA- and LPS-stimulated splenocytes by 48.17% and 31.75%, respectively (*p* = 0.0006; *p* = 0.0296). This inhibitory effect was partly counteracted upon the addition of the GR antagonist RU486. The proliferation capacity under RU486 intervention increased by 200.54% and 215.65% (both *p* < 0.0001) relative to the CORT group, exceeding the levels observed in the untreated LD group (Figure 8A,B), which is consistent with a contributory role for GR-dependent signalling in CORT-associated suppression of splenocyte activation.

Associated changes in intracellular signalling pathways were subsequently analysed. Western blot results indicated that CORT treatment was associated with increased GR protein expression, with increases of 63.70% and 154.56% in ConA- and LPS-stimulated cohorts, respectively (*p* = 0.0161; *p* = 0.0016). The addition of RU486 partly counteracted this pattern, causing GR protein expression to decline by 35.58% and 52.25% in T and B cells, respectively (*p* = 0.0241; *p* = 0.0035), relative to the CORT group (Figure 8C,D). Changes in GR expression occurred alongside alterations in NF-κB-related inflammatory signalling.CORT exposure led to 92.47% and 77.70% increases in p-p65 phosphorylation in ConA- and LPS-stimulated splenocytes, respectively (*p* = 0.0005; *p* = 0.0001). However, RU486 significantly reduced this activation, reducing the phosphorylation levels by 64.46% and 55.68% in these respective cell types (*p* < 0.0001; *p* = 0.0007). The GR and p-p65 profiles suggest a link between the CORT-GR axis and enhanced NF-κB-related inflammatory signalling.

Concurrently, CORT treatment was associated with reduced Nrf2/HO-1-associated antioxidant responses. CORT exposure was associated with 63.39% and 54.94% reduction in Nrf2 expression in ConA- and LPS-stimulated splenocytes (*p* = 0.0022; *p* = 0.0003), while HO-1 levels were reduced by 53.50% and 51.56% (*p* = 0.0001; *p* < 0.0001). However, this defence system increased following RU486 treatment. In both ConA- and LPS-stimulated splenocytes, Nrf2 expression increased by 163.12% and 72.03%, respectively (*p* = 0.0003; *p* = 0.0052), while HO-1 levels increased by 164.65% and 151.58%, respectively (*p* < 0.0001; *p* = 0.0052), approaching baseline levels (Figure 8G–J).

Finally, changes in the protein expression of the proliferation marker PCNA further reflected the cellular proliferation-related profile. CORT exposure decreased PCNA abundance in ConA- and LPS-stimulated cohorts by 64.14% and 49.68%, respectively (*p* = 0.0005; *p* = 0.0041), whereas RU486 treatment increased these levels by 43.60% and 61.03% relative to the CORT-treated cells (*p* = 0.0040; *p* = 0.0364), respectively (Figure 8K,L). This PCNA expression pattern closely aligns with the observed trends in the stimulation index, suggesting that CORT-associated suppression of cellular proliferation may involve GR-related modulation of downstream inflammatory and antioxidant signalling.

Overall, these in vitro findings are consistent with the in vivo observations, indicating that CORT can modulate primary splenocyte activation via GR-dependent signalling. Taken together, the in vitro data support a contributory role for the CORT-GR axis in linking stress signalling with splenic immune alterations, while also recognising the complex multi-pathway nature of the in vivo .

### 3.6. Transcriptomic Analysis Supports Partial Reversal of the dBL-Associated Inflammatory and Immunosuppressive Transcriptional Profile After CORT Blockade

To assess the association between corticosterone signalling and splenic gene-expression patterns at a broader systems level, RNA-Seq analysis was performed on spleen tissue from the LD, dBL, and BMet cohorts. Principal component analysis (PCA) and Venn diagram analysis showed separation among the three experimental groups, indicating treatment-associated differences in the transcriptional landscape (Figure S3A,B).

**Figure 9:**
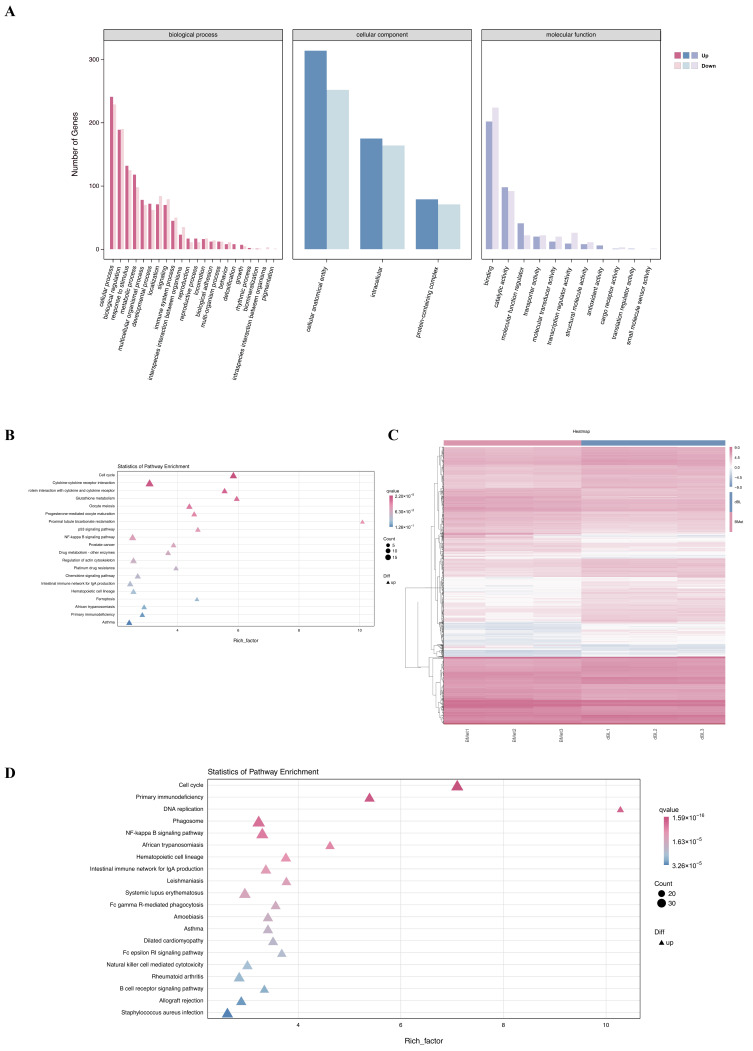
**Transcriptomic analysis following CORT synthesis blockade.** (**A**) LD vs. dBL gene ontology (GO) enrichment analysis. (**B**) LD vs. dBL KEGG pathway analysis. (**C**) dBL vs. BMet gene clustering heatmap. (**D**) dBL vs. BMet KEGG pathway analysis. Biological replicates were used for all sequencing analyses (*n* = 3). Differentially expressed genes were identified using FDR < 0.01, *p* < 0.01, and an absolute fold change ≥ 2, as described in the Methods. Sequencing depth was evaluated based on the number of clean reads and clean bases generated for each sample. The per-sample clean reads, clean bases, GC content, and Q30 values are provided in Supplementary Table S3.

Transcriptomic comparisons between the LD and dBL groups revealed extensive differential gene expression (Figure S3C). Gene Ontology (GO) enrichment analysis underscored that these dBL-regulated genes predominantly clustered within functional categories such as immune system processes, stress responses, and regulation of cell death (Figure 9A). Furthermore, KEGG pathway analysis revealed that in the dBL cohort, pro-inflammatory pathways such as the NF-κB signalling pathway were highly enriched, whilst the significant upregulation of pathways like ‘Primary immunodeficiency’ indicated altered immune-related trascriptional activity. These results provide transcriptomic evidence that dBL exposure was associated with a pro-inflammatory and immunosuppressive splenic transcriptional profile (Figure 9B).

To further examine whether corticosterone blockade was associated with changes in this transcriptional profile, differentially expressed genes between the dBL and BMet groups were analysed. Gene clustering heatmap analysis and volcano plots (Figure S3D) showed that BMet treatment partially shifted the expression profile away from the dBL-associated pattern (Figure 9C). KEGG enrichment analysis similarly identified the NF-κB signalling pathway. Compared with the dBL group, BMet treatment was associated with attenuation of several pro-inflammatory transcriptional changes and a partial shift in immune-function-related gene expression patterns (Figure 9D).

**Figure 10:**
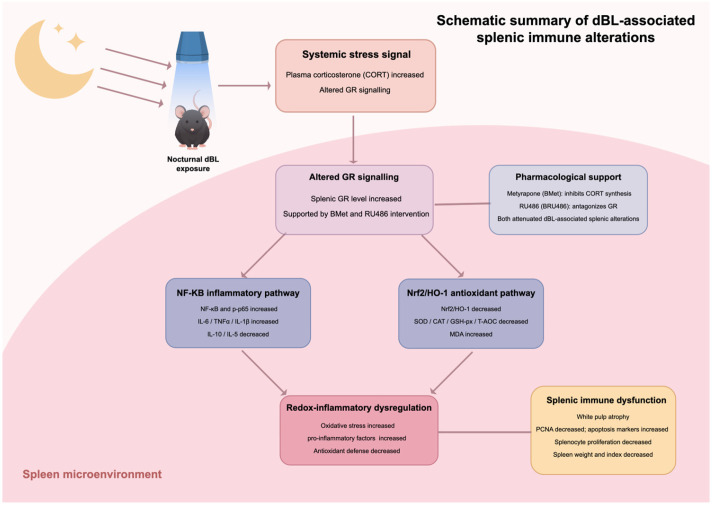
**Schematic summary of dBL-associated splenic immune dysfunction**. Nocturnal dBL exposure in HFD-fed mice was associated with elevated plasma corticosterone, altered splenic GR signalling, increased NF-κB-related inflammatory signalling, and reduced Nrf2/HO-1-associated antioxidant responses. Pharmacological modulation with metyrapone or RU486 partially attenuated several dBL-associated splenic alterations, which is consistent with the involvement of CORT-GR signalling. Created with Figdraw (https://www.figdraw.com/).

Taken together, transcriptomic evidence supports an association between nocturnal dBL exposure, altered corticosterone signalling, and changes in the splenic transcriptional landscape. Inhibition of corticosterone synthesis with Metyrapone was associated with partial attenuation of the pro-inflammatory and immunosuppressive transcriptional profile, providing supportive systems-level evidence for the involvement of corticosterone signalling in dBL-associated splenic immune alterations. A schematic summary of these dBL-associated splenic immune alterations and the proposed involvement of CORT-GR signalling is presented in Figure 10.

## 4. Discussion

Current lifestyles are increasingly characterised by the dual challenges of dietary imbalances and environmental light pollution. To simulate this prevalent physiological context, the present investigation established a nocturnal exposure model using monochromatic treatments of varying wavelengths in mice maintained on a high-fat diet (HFD). It was observed that although each monochromatic light group simulated unnatural illumination conditions, the biological effects elicited exhibited wavelength specificity. The present data indicate that, among the light spectra tested, dim blue light (dBL) exposure was associated with the most pronounced splenic alterations, reduced antioxidant responses, and a pro-inflammatory cytokine imbalance. This oxidative and inflammatory microenvironment was accompanied by disrupted apoptotic homeostasis and reduced splenic cellularity and proliferative capacity. This cellular loss was reflected in histopathological atrophy of the white pulp and overall organ regression. In contrast, dim red light (dRL) or dim green light (dGL) displayed negligible disparities in the aforementioned oxidative and inflammatory markers, with spleen morphology remaining relatively stable. This comparative analysis indicates that the splenic responses differed according to wavelength. Specifically, dBL, characterised by its high single-photon energy within the 400–480 nm range, was the light condition most closely associated with splenic immune alterations in the present HFD-fed model [44,45,46,47]. These findings suggest that short-wavelength nocturnal light is particularly relevant to neuroendocrine-immune stress responses.

Our findings align with and substantially extend the growing body of evidence regarding the systemic impacts of artificial light at night (ALAN). Previous investigations have established that generalised nocturnal light exposure acts as a potent environmental stressor capable of activating the hypothalamic–pituitary–adrenal (HPA) axis, frequently resulting in elevated circulating corticosterone levels and disrupted diurnal endocrine rhythms [1,48]. Concurrently, broader ALAN models have been shown to disrupt cellular redox homeostasis, precipitating oxidative stress through the accumulation of reactive oxygen species and the depletion of endogenous antioxidants across various peripheral tissues [49,50]. From an immunological perspective, prolonged light pollution is closely associated with systemic inflammation and altered cytokine profiles, ultimately leading to immunosuppression or the functional dysregulation of peripheral lymphoid organs [51,52]. While these broader physiological connections have been well documented, our study adds evidence for wavelength-dependent differences in splenic responses under a uniform HFD background. Specifically, our findings suggest that dBL was the light condition more closely associated with this neuroendocrine-oxidative-immune profile in the present HFD-fed model.

Building upon these phenotypic observations, prolonged exposure to dBL appears to be associated with a systemic stress-related response involving the neuroendocrine-immune axis [53,54]. The involvement of this non-visual pathway in endocrine homeostasis provides a plausible framework for interpreting how wavelength-specific nocturnal light may be associated with adverse alterations in peripheral immune organs [55,56,57]. Based on the phenotypic disparities observed, dBL was associated with a greater physiological burden than the other wavelengths tested under the present HFD-fed condition. These findings support the view that spectral composition should be considered alongside illuminance when assessing the biological effects of ALAN [58,59,60,61].

Although the present findings suggest an association between dBL and immune function during the night, the light intensity was calibrated based on human visual illuminance (lux). Due to the inherently lower luminous efficiency of short-wavelength light, achieving 5 lx of monochromatic dBL requires a markedly higher absolute irradiance than 5 lx of broad-spectrum dWL [2,62]. Consequently, the pronounced immune impairment observed in the dBL group may partly relate to the greater radiometric power required to achieve equivalent photopic illuminance with short-wavelength light. This also suggests that environments dominated by the blue light spectrum, such as electronic screens or urban night lighting, exert physiological impacts that are disproportionate to visual perception, even under ambient light conditions perceived as equally dim by the human eye [63].

Furthermore, although dWL in this study also contained the blue light spectrum, it did not exhibit significant adverse effects, suggesting the possible existence of buffering mechanisms within the retinal neural circuitry. Current evidence indicates that long-wavelength light inputs (e.g., red and green light) can antagonise intrinsically photosensitive retinal ganglion cells (ipRGCs) that express melanopsin, which are involved in non-visual photic signal transduction [64,65]. Broad-spectrum dWL may have engaged retinal antagonistic pathways that attenuated the downstream neuroendocrine signal. In contrast, monochromatic dBL may provide a less counterbalanced short-wavelength signal, which could contribute to altered CORT-GR signalling and splenic immune changes.

Our pathway-level analyses suggest that nocturnal dBL exposure is associated with altered CORT secretion and splenic GR signalling, which may contribute to downstream redox-inflammatory imbalance and remodelling of the immune microenvironment. Previous studies indicate that sustained glucocorticoid exposure mediates immunosuppression through GR [66,67], which is highly expressed in the spleen and acts as an important endocrine-immune transducer for environmental photostress [68,69]. GR-related signalling has been reported to interact with NF-κB and Nrf2/HO-1 pathways, potentially contributing to inflammatory activation and reduced antioxidant responses [70,71]. In the present study, CORT-GR appeared to contribute to these alterations, as pharmacological modulation partially attenuated oxidative stress and inflammatory infiltration [72,73,74]. Specifically, the observed CORT elevation was accompanied by reduced PCNA abundance, consistent with reduced splenocyte proliferative capacity and immune surveillance. By showing that in vivo BMet/RU486 intervention partly improved splenic microarchitecture and mitogen-induced proliferative responses and activation capacities, our findings provide pharmacological support for the CORT-GR axis as a contributory pathway in environmental light pollution-associated stress [75,76,77]. However, considering the partial nature of the pharmacological attenuation observed in our study and the inherent complexity of the neuroendocrine-immune network, CORT-GR signalling may act alongside other systemic mediators rather than in isolation. Additional neuroendocrine pathways may also contribute to the observed splenic phenotype. For instance, the sympathetic nervous system (SNS) directly innervates the spleen, and light-associated sympathetic hyperactivation can influence local immune responses via adrenergic signalling [78,79]. Concurrently, nocturnal light exposure may suppress pineal melatonin synthesis. Given melatonin’s endogenous antioxidant and immunomodulatory properties, its depletion may exacerbate systemic oxidative stress and limit the organism’s buffering capacity against light-associated stress [80,81]. Therefore, the splenic immune alterations reported here should be viewed as a composite outcome of a multifaceted neuroendocrine dysregulation, wherein CORT-GR signalling appears to be one contributory component within this broader response.

The interpretation that CORT-GR signalling is involved in these splenic changes is consistent with convergent evidence from several experimental levels. In the primary exposure cohort, dBL exposure under an HFD background was accompanied by elevated circulating CORT, altered splenic GR expression, increased NF-κB-related signalling, reduced Nrf2/HO-1-associated antioxidant responses, and impaired splenocyte proliferative capacity. In an independent pharmacological cohort, inhibition of CORT synthesis or GR signalling attenuated several of these inflammatory, oxidative, structural, and functional alterations. In primary splenocytes, CORT treatment suppressed cellular activation, while RU486 partly reversed these responses. Transcriptomic analysis further provided supportive evidence that CORT blockade was associated with partial attenuation of an inflammatory and immunosuppressive transcriptional profile. Taken together, these findings support the involvement of CORT-GR signalling but do not establish this pathway as the sole or definitive mechanism underlying the phenotype.

In terms of systemic implications, the wavelength-dependent differences observed in this study may provide useful evidence for assessing nocturnal light exposure in contemporary occupational and urban environments. These findings suggest that reduce short-wavelength light exposure at night, or preserving neuroendocrine homeostasis, may be relevant to mitigating systemic immune disruption[82]. Consistent with this interpretation, transcriptomic data provide supportive systems-level evidence that corticosterone signalling blockade is associated with partial reversal of the splenic pro-inflammatory and immunosuppressive transcriptional profile [83,84].

While this study provides evidence that CORT-GR signalling is involved in dBL-associated splenic alterations, several limitations should be noted. Because all experimental animals were maintained on an HFD, the present design cannot distinguish the independent effects of dBL from those of diet, nor can it formally test an interaction between diet and light. Therefore, all conclusions should be interpreted within the HFD-fed model, and causal attribution to nocturnal light exposure alone should be avoided. Another limitation is the exclusive use of male mice. Given that glucocorticoid signalling and systemic immune responses are influenced by sex hormones and the oestrous cycle, our findings cannot be directly generalised to females. Future studies should incorporate female cohorts to examine potential sexual dimorphism in response to wavelength-specific photic stress. In addition, this work primarily examined a fixed exposure period. The potential cumulative impact of longer-term light exposure on immunological balance warrants further investigation. Furthermore, as our transcriptomic and molecular analyses were performed on tissues collected at the end of the 12-week exposure, they provide a terminal snapshot of the biological state. Although the independent pharmacological cohort supports the involvement of CORT-GR signalling, the lack of longitudinal assessment limits our ability to establish temporal causal relationships. Finally, the absence of an a priori power analysis for sample size determination is a limitation. The main morphological, molecular, and cellular functional analyses were conducted with five biological replicates per group, while cell protein-related Western blot analyses and transcriptomic sequencing were conducted with three biological replicates per group. These smaller-sample analyses were used as supportive evidence and interpreted alongside convergent findings from independent experimental approaches.

Moreover, because our environmental model explicitly relies on the framework of circadian disruption, the absence of temporal expression profiling for canonical molecular clock genes, such as *Bmal1*, *Clock*, *Per1/2*, and *Cry1/2*, is an acknowledged shortcoming. Given the inherent 24 h oscillatory nature of core clockworks, alongside the endocrine, oxidative stress, and immune networks they govern, accurately characterising their dysregulation, such as phase shifts or amplitude dampening, would require multi-time-point tissue sampling rather than a single-time-point snapshot at ZT0 (08:00). Thus, while standardising tissue collection at this specific diurnal transition effectively minimised circadian-induced variance to ensure reliable cross-group comparability, our current mechanistic exploration remains limited to the downstream neuroendocrine effectors, namely the CORT-GR axis, and immunological endpoints at a single temporal cross-section. In parallel, while the present study captured the global redox-inflammatory shift within the splenic microenvironment, the lack of flow cytometric phenotyping restricts our cellular insights. Future studies should include detailed single-cell immune profiling to precisely dissect how photic stress distinctively skews specific lymphocyte and macrophage subpopulations, including T/B cell ratios and macrophage polarisation. To complement this cellular perspective, high-resolution time-course circadian transcriptomics should also be employed to fully map the local splenic clockwork under light pollution, aligning with recent authoritative paradigms in circadian biology [85].

Finally, beyond the spleen, the involvement of other systemic lymphoid tissues, such as the thymus and bone marrow, in cumulative circadian disturbances warrants holistic multi-organ assessment to construct a more complete picture of light-associated immune alterations. Ultimately, the present findings support the consideration of spectral composition in environmental lighting assessment, particularly in relation to neuroendocrine-immune homeostasis.

## Data Availability

The original contributions presented in this study are included in the article/Supplementary Materials. Further inquiries can be directed to the corresponding author.

## Supplementary Materials

The following supporting information can be downloaded at: https://www.mdpi.com/article/10.3390/antiox15070800/s1, Figure S1: Nocturnal dBL-associated splenic transcriptional alterations under an HFD background. Figure S2: Pharmacological intervention-associated changes in dBL-related splenic transcriptional alterations. Figure S3: Additional transcriptomic characterisation following CORT synthesis blockade. Table S1: Nutritional composition and ingredient formulation of the high-fat diet (HFD). Table S2: Sequences of primers used for RT-qPCR. Table S3: Summary of RNA-seq data quality.
