# Supplementary material for: Nocturnal Dim Blue Light Is Associated with Splenic Immune Dysregulation and Altered CORT-GR Signalling in High-Fat-Diet-Fed Mice
Source: Antioxidants (Basel). 2026 Jun 26;15(7):800. doi: 10.3390/antiox15070800 (PMC13403523; doi:10.3390/antiox15070800)
Supplement: Supplementary file 1 [file antioxidants-15-00800-s001.zip › antioxidants-4336594-supplementary.pdf]

Supplementary Figures

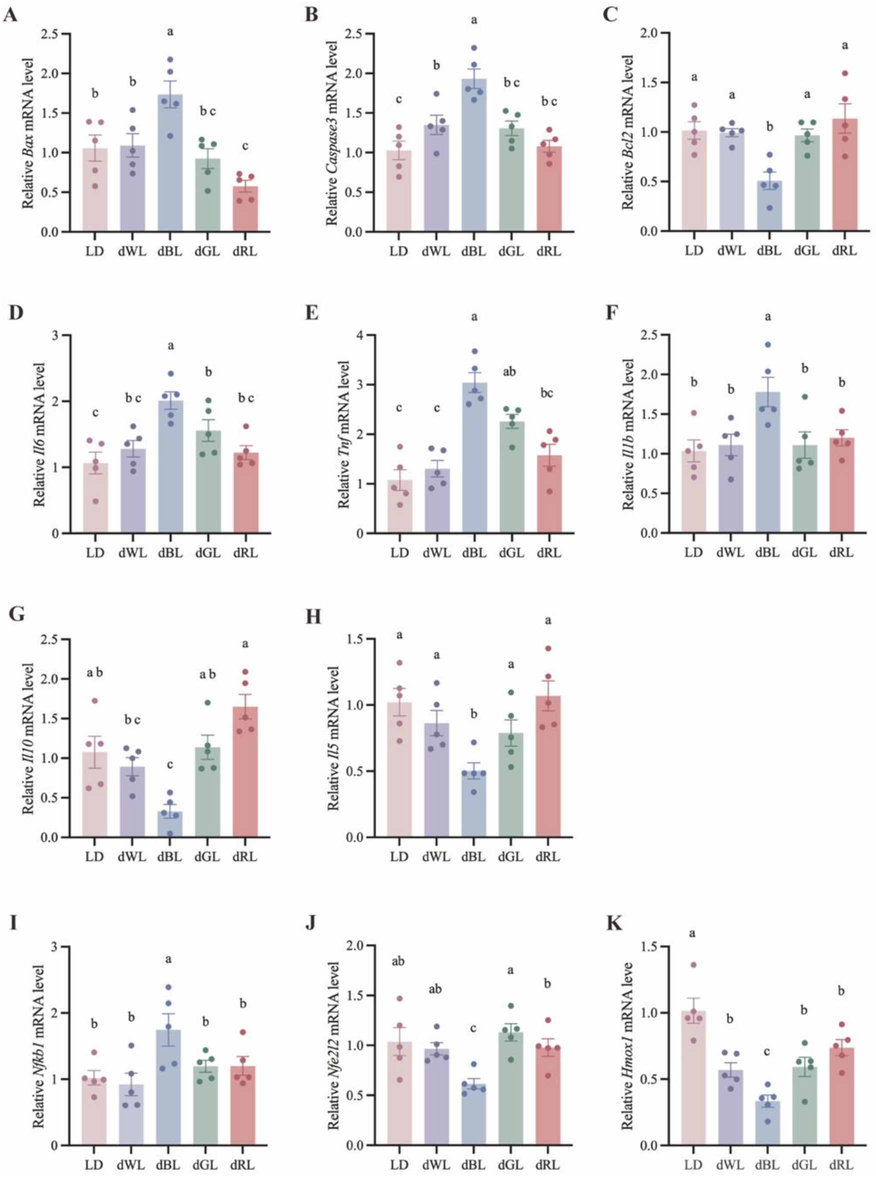

Figure S1. Nocturnal dBL-induced splenic inflammatory responses.

(A–C) Relative mRNA expression levels of *Bax*, *Caspase-3*, and *Bcl-2* in the spleen. (D–H) Relative mRNA expression of *Il6*, *Tnf*, *Il1b*, *Il10*, and *Il5*. (I–K) Relative mRNA levels of *Nfkb1*, *Nfe2l2* and *Hmox1*. Data are presented as mean ± SEM (n = 5) and were analysed via one-way ANOVA. Different letters (a, b, c, d) indicate significant differences among groups ($P < 0.05$), with exact *P*-values provided in the

corresponding Results section. LD: control group exposed to the standard light/dark cycle; dWL: dim white light group; dBL: dim blue light group; dGL: dim green light group; dRL: dim red light group.

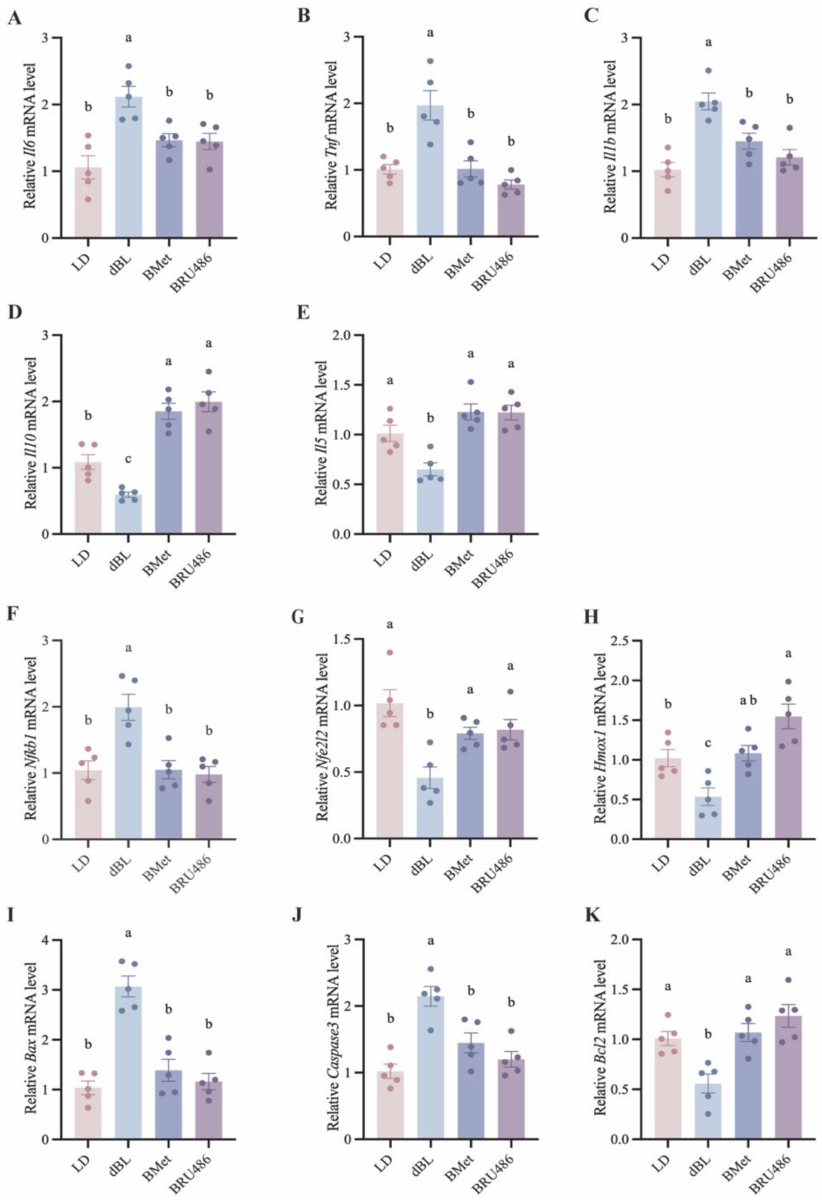

Figure S2. Alterations in dBL-induced splenic inflammatory responses following pharmacological intervention.

(A–E) Relative mRNA expression of *Il6*, *Tnf*, *Il1b*, *Il10*, and *Il5*. (F–H) Relative mRNA levels of *Nfkb1*, *Nfe2l2* and *Hmox1*. (I–K) Relative mRNA expression levels of *Bax*, *Caspase-3*, and *Bcl-2* in the spleen. Data are presented as mean ± SEM (n = 5). Statistical significance was determined as described in Figure S1. LD: control group exposed to the standard light/dark cycle; dBL: dim blue light group; BMet: dim blue light with metyrapone group; BRU486: dim blue light with RU486 group.

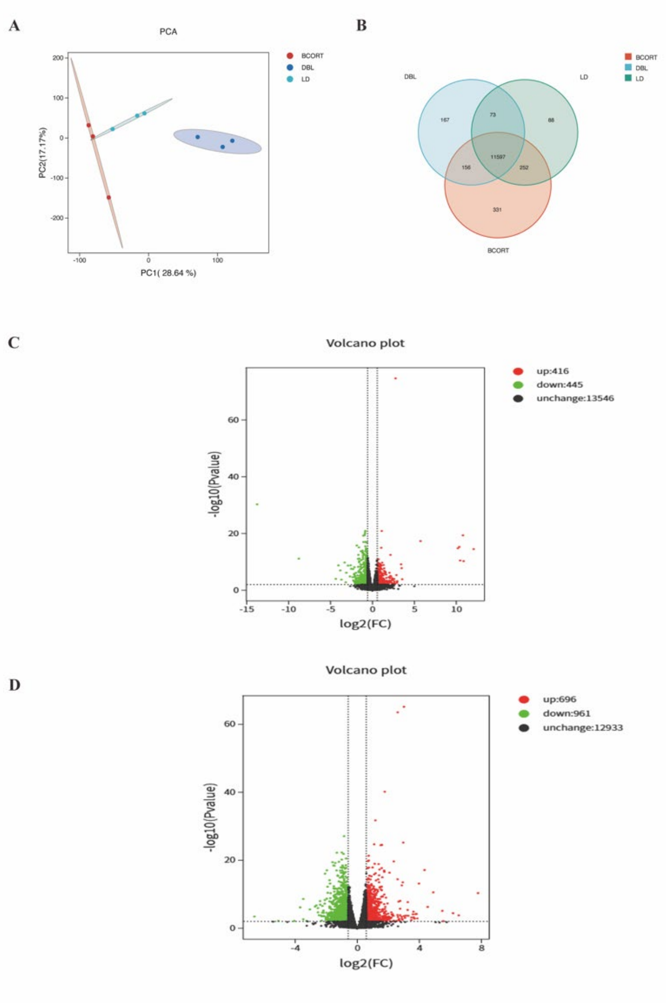

Figure S3. Transcriptomic validation of CORT blockade.

(A) Principal component analysis (PCA). (B) Venn diagram. (C) LD vs. dBL volcano plot. (D) dBL vs. BMet volcano plot. The 'BCORT' within the figure panels

corresponds to the BMet treatment group. Transcriptomic analyses were performed with 3 biologically independent samples per group (n = 3). Differentially expressed genes were defined according to the strict statistical thresholds detailed in the Methods section.

Supplementary Table

Table S1. Supplementary Table S1. Nutritional composition and ingredient formulation of the high-fat diet (HFD)

**Product**	**H10060**	
	**gm%**	**kcal%**
Protein	26	20
Carbohydrate	26	20
Fat	35	60
Total		100
kcal/gm	5.24	
**Ingredients:** Lard, rich in saturated fatty acids, serves as the primary dietary fat source. The formulation primarily consists of casein, dextrin, sucrose, cellulose, soybean oil, mineral and vitamin mixes, and choline.		

Table S2. Sequences of primers used for RT-qPCR

Genes	Primer sequence (5' to 3')	Accession No.
Il5	F: GACTTCAGAGTCATGAGAAGGAT	NM_010558.1
	R: GACAGGAAGCCTCATCGTCT	
Il6	F: CTGCAAGAGACTTCCATCCAG	NM_001314054.1
	R: AGTGGTATAGACAGGTCTGTTGG	
Il1b	F: GAAATGCCACCTTTTGACAGTG	NM_008361.4
	R: TGGATGCTCTCATCAGGACAG	
Il10	F: CTTACTGACTGGCATGAGGATCA	NM_010548.2
	R: GCAGCTCTAGGAGCATGTGG	
Tnf	F: CAGGCGGTGCCTATGTCTC	NM_001278601.1
	R: CGATCACCCCGAAGTTCAGTAG	
Bcl2	F: TCTTTGAGTTCGGTGGGGTC	NM_009741.5
	R: TTTGTTTGGGGCAGGTTTGTC	
Bax	F: GCTGGACACTGGACTTCCTC	NM_007527.4

	R: GGGGTCCCGAAGTAGGAGAG	
Caspase-3	F: GAGCTTGGAACGGTACGCTAA	NM_001284409.1
	R: CGTCCACATCCGTACCAGAG	
Nfkb1	F: AGGCTTCTGGGCCTTATGTG	NM_009045.5
	R: TGCTTCTCTCGCCAGGAATAC	
Nfe2l2	F: TCTTGGAGTAAGTCGAGAAGTGT	NM_010902.5
	R: GTTGAAACTGAGCGAAAAAGGC	
Hmox1	F: AAGCCGAGAATGCTGAGTTCA	NM_010442.2
	R: GCCGTGTAGATATGGTACAAGGA	
GRα	F: TTCGCAGGCCGCTCAGTGTT	XM_036160990.1
	R: AGGTGGTCCCGTTGCTGTGGA	
GRβ	F: AAAGAGCTAGGAAAAGCCATTGIC	NC_000084.7
	R: CTGTCTTTGGGCTTTTGAGATAGG	
Nr3c1	F: TGAAGCTTCGGGATGCCATT	XM_036160990.1
	R: ATTGTGCTGTCCTTCCACTG	
GAPDH	F: CCGAGAATGGGAAGCTTGTC	NM_ 001289726.1
	R: TTCTCGTGGTTCACACCCATC	

F = Forward primer; R = Reverse primer.

Table S3. Summary of RNA-seq data quality

Sample	Clean reads	Clean bases	GC Content	%≥Q30
LD1	22,624,073	6,743,683,622	48.18%	95.70%
LD2	23,295,892	6,955,942,664	48.90%	95.45%
LD3	21,210,768	6,333,229,996	48.61%	95.49%
dBL1	23,067,284	6,890,491,836	48.66%	95.40%
dBL2	22,473,978	6,713,021,174	48.14%	95.50%
dBL3	22,468,738	6,704,003,257	47.33%	95.66%
BMet1	19,564,267	5,841,533,476	48.10%	95.80%
BMet2	21,482,421	6,404,462,718	48.01%	95.60%
BMet3	22,068,034	6,593,196,663	48.27%	95.63%
